# Assessment of Prior Infection With Hepatitis B Virus and Fecundability in Couples Planning Pregnancy

**DOI:** 10.1001/jamanetworkopen.2023.30870

**Published:** 2023-08-31

**Authors:** Jun Zhao, Yan Xuan, Yue Zhang, Xiang Hong, Hongguang Zhang, Rong Zhang, Tao Yan, Yuanyuan Wang, Zuoqi Peng, Ya Zhang, Kailei Jiao, Tianyu He, Qiaomei Wang, Haiping Shen, Yiping Zhang, Donghai Yan, Bei Wang, Xu Ma

**Affiliations:** 1 National Research Institute for Family Planning, Beijing, China; 2 National Human Genetic Resources Center, Beijing, China; 3 Key Laboratory of Environmental Medicine and Engineering of Ministry of Education, Department of Epidemiology and Statistics, School of Public Health, Southeast University, Nanjing, Jiangsu, China; 4 Department of Maternal and Child Health, National Health Commission of the People’s Republic of China, Beijing, China

## Abstract

**IMPORTANCE:**

The association of hepatitis B virus (HBV) infection with reduced fecundability among reproductive-aged couples lacks large-population, in-depth study evidence.

**OBJECTIVE:**

To investigate the association of HBV infection with time to pregnancy in couples planning pregnancy, and to explore whether this association varied by gravidity, health statuses, or lifestyles.

**DESIGN, SETTING, AND PARTICIPANTS:**

This is a population-based cohort study of Chinese couples participating in the National Free Preconception Check-up Projects during 2015 to 2017. They were planning pregnancy and were followed-up every 3 months until getting pregnant, as confirmed by gynecologic ultrasonography, or were followed-up for 1 year. Data were analyzed between March 1, 2022, and September 30, 2022.

**MAIN OUTCOMES AND MEASURES:**

The main outcome was time to pregnancy, assessed using fecundability hazard ratios (HRs). The Cox proportional hazards regression models were used to estimate the association of HBV infection with fecundability.

**RESULTS:**

Among 2 419 848 couples (mean [SD] age, 27.87 [5.20] years for women and 29.58 [5.50] years for men), 126 728 women (5.24%) and 156 572 men (6.47%) were infected with HBV. Compared with the HBV-negative group, the fecundability of both women and men in the HBV-positive group decreased by 5% (HR, 0.95; 95% CI, 0.94-0.95). Compared with couples in which both partners were HBV negative, the fecundability of those in which both partners were HBV positive declined by 6% (HR, 0.94; 95% CI, 0.93-0.96) among all couples, by 3% (HR, 0.97; 95% CI, 0.95-0.99) among nulligravidas couples, and by 7% (HR, 0.93; 95% CI, 0.91-0.95) among multigravidas couples. Both the female-male and couple models suggested that the association of HBV infection with decreased fecundability was more pronounced in couples with multigravidas. The negative association was greater in people with overweight and obesity and was inconsistent in certain subgroups; in particular, it was more pronounced in women with reproductive tract infections, normal fasting plasma glucose, and no alcohol intake and in men with normal blood pressure.

**CONCLUSIONS AND RELEVANCE:**

In this population-based cohort study, HBV infection was associated with decreased fecundability in a general reproductive-aged population, especially in couples with multigravidas. For women and men with certain health statuses and lifestyles, a comprehensive consideration of this association is recommended to provide personalized fertility guidance.

## Key Points

**Question:**

Is hepatitis B virus (HBV) infection associated with reduced fecundability in reproductive-aged couples, and does this association vary by population characteristics?

**Findings:**

This large population-based cohort study of 2 419 848 couples in China assessed the fecundability of couples in which both spouses had HBV infection and found that HBV infection was associated with reduced fecundability. This association was more pronounced in couples with multigravidas and in people with overweight and obesity.

**Meaning:**

The association of HBV infection with fecundability should be comprehensively considered for couples planning pregnancy, especially those with certain health statuses and lifestyles.

## Teaser

This cohort study investigates the association of hepatitis B virus infection and time to pregnancy in couples planning pregnancy and explores whether this association varies by gravidity, health status, or lifestyle.

## Introduction

Infertility, which is defined by the World Health Organization as the failure to conceive within 12 months of regular unprotected sexual intercourse, has drawn global concern.^1,2^ With the increasing economic, mental, and social pressures, the prevalence of infertility has reached an average incidence of 12.5% globally and is still increasing.^3^ In practice, time to pregnancy (TTP) is an effective and widely used indicator that can be used to evaluate fecundability.^4^ Prolonged TTP generally indicates subfertility or low fecundability.^5^ Meanwhile, hepatitis B virus (HBV) infection is a major global public health threat, infecting approximately 296 million people worldwide in 2019.^6^ China has the largest population living with HBV, contributing to one-third of the world’s total.^7^ Research^8,9^ has found that HBV not only invades human the liver, but also can be detected in semen and follicles of infected persons. Population studies^10,11^ have found that HBV infection is associated with increased risk of female tubal infertility and is related to longer durations of infertility and lower rates of implantation among patients undergoing in vitro fertilization treatment.^12^ Nevertheless, the association of HBV infection with human fecundability has not been conclusively established.^9,10,12,13,14,15^

Previous studies^9,10,11,12,13,14,15^ on HBV infection and fecundability were conducted in infertile populations, and, to our knowledge, no large sample studies have been conducted in the general reproductive-aged population. Because of the specificity of infertile patients and limited sample size, previous studies^9,11^ did not evaluate the fecundability of couples in which both partners had HBV infection, nor did they explore associations between them in populations with different characteristics. Therefore, we conducted a large, pregnancy-planning cohort study based on the National Free Pre-conception Check-up Project (NFPCP) to investigate whether HBV infection was associated with decreased fecundability in reproductive-aged couples in the general population, to assess the fecundability of couples in which both spouses have HBV infection, and to further analyze the association of HBV infection with fecundability in couples with different gravidity, health statuses, and lifestyles, so as to provide personalized fertility guidance for couples with different characteristics.

## Methods

### Study Participants

The NFPCP was a nationwide population-based cohort study initiated by the Chinese government to reduce the incidence of adverse pregnancy outcomes. Detailed information about the protocols, design, organization, and implementation had been described previously.^16^ We used NFPCP data from January 1, 2015, to December 31, 2017, involving 2 564 909 eligible Chinese married couples. After signing written informed consent, they completed prepregnancy examinations and risk assessments, and pregnancy outcomes were followed-up by telephone at 3-month intervals until pregnancy or at least 1 year. All couples were not pregnant when participating and expressed they were ready for pregnancy. To be included in the study, couples’ pregnancy outcomes had to be followed for at least 1 year, couples had to ensure that they were not pregnant when enrolled, and the results of HBV serological indexes for both spouses all had to be negative or hepatitis B surface antigen (HBsAg)–positive. Male partners had to be at least 22 years old, and female partners had to be aged 20 to 49 years during participation.

**Figure 1.**
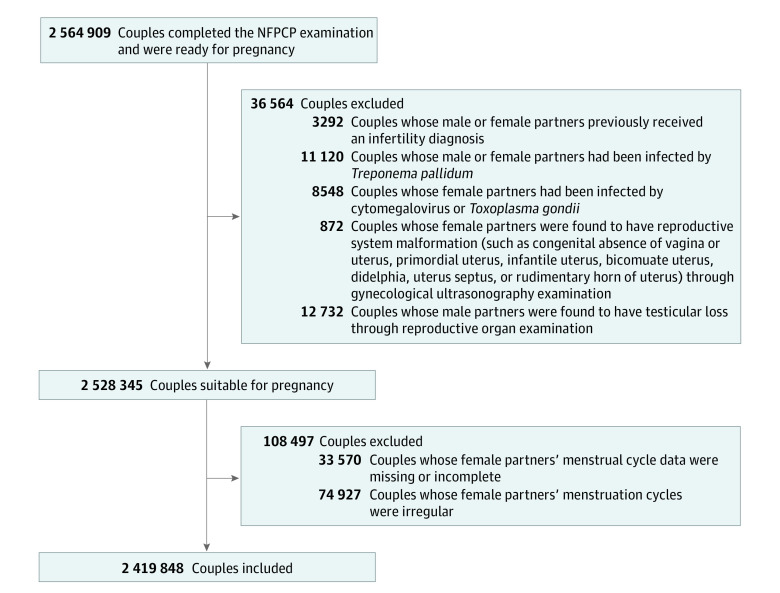
Flowchart for the Study Population NFPCP indicates National Free Pre-conception Check-up Project.

According to the exclusion criteria, couples were excluded if they had specific diseases that caused difficulties or risks to conceive presently, or if female partners lacked menstrual information or had irregular menstruation (ie, a cycle with an intermenstrual interval of <21 days or ≥35 days or the variation of cycle length from 1 period to another was >7 days^17^) (Figure 1). This study was approved by the institutional research review board at the National Health Commission and the National Health Council’s Ethics Review Committee, and it followed the Strengthening the Reporting of Observational Studies in Epidemiology (STROBE) reporting guideline.

### Study Procedure

Trained medical staff from maternal and child health institutions collected participants’ self-reported sociodemographic characteristics (including age, ethnicity, educational level, occupation, and region) and menstrual and reproductive history (including the number of children in their current family, pregnancy history, age at menarche, menstrual period length, and menstrual cycle length) through standardized questionnaires administered by face-to-face interview. Participants’ health status and lifestyles (including body mass index [BMI] calculated as weight in kilograms divided by height in meters squared, alcohol intake, tobacco exposure, hypertension, reproductive tract infections, fasting plasma glucose, and contraception) were obtained by questionnaires, physical examinations, or laboratory tests.

According to the NFPCP standard protocol, participant’s height (nearest 0.1 cm) and weight (nearest 0.1 kg) were measured barefoot, without coats and shoes, and resting blood pressure was measured from participant’s right arm using an automatic sphygmomanometer after resting for at least 10 minutes. Blood samples were collected after fasting for at least 8 hours. HBV serological indexes were determined by enzyme-linked immunosorbent assay, and fasting plasma glucose was measured by the glucose oxidase method. Gynecologists collected vaginal secretion swabs by gently swabbing the vaginal vault, performed leucorrhea examinations, and microscopically examined the genital tract pathogens. All couples underwent reproductive system examinations to exclude genital malformations and other abnormalities affecting fecundability. All results were obtained strictly following the NFPCP quality assurance protocol, and the laboratory quality was monitored and supervised by the National Center of Clinical Laboratories for Quality Inspection and Detection.

### Exposure Measurements

HBV serological indexes included HBsAg, hepatitis B virus surface antibody, hepatitis B e antigen, hepatitis B virus e antibody, and hepatitis B core antibody. We defined being positive for HBsAg as having HBV infection (HBV positive), and being negative for HBsAg, hepatitis B virus surface antibody, hepatitis B e antigen, hepatitis B virus e antibody, and hepatitis B core antibody as not having HBV infection (HBV negative).

### Ascertainment of Outcome

The main outcome was TTP, calculated according to clinical pregnancy results confirmed by gynecologic ultrasonography in women. TTP was defined as the interval between the last menstruation date (for pregnant couples) or the last follow-up date (for nonpregnant couples) obtained at last follow-up and the last menstruation date obtained at baseline. Among women with regular menstruation, TTP in cycles for each woman was calculated separately by the TTP divided by the average menstrual cycle length of her last 6 cycles, whereas TTP in months was calculated by the TTP divided by 30 among all women.^18,19^ TTP was considered censored if the follow-up was longer than 1 year and couples were still not pregnant.

### Covariates Definition

The covariates in this study were selected on the basis of previous studies^16,20,21,22,23,24,25,26,27,28,29,30^ related to TTP or subfertility. Region referred to the province where the inspection agency was located, divided into eastern, central, and western.^31^ BMI was calculated and then categorized into 4 groups^32^: underweight (<18.5), normal weight (18.5-23.9), overweight (24.0-27.9), and obesity (≥28). Alcohol intake was defined as drinking at least once per week, regardless of the amount of alcohol.^33^ Tobacco exposure was defined as either active smoking (smoking ≥1 cigarette per day for at least 1 year) or passive smoking (daily exposure to environmental tobacco smoke) during study participation.^21^ Hypertension referred to systolic blood pressure greater than or equal to 140 mm Hg and/or diastolic blood pressure greater than or equal to 90 mm Hg.^32^ Reproductive tract infection meant at least 1 of the following pathogens—*Gardnerella vaginalis*, *Trichomonas vaginalis*, *Candida* species, *Neisseria gonorrhoeae*, or *Chlamydia trachomati*—was detected in vaginal secretions. Fasting plasma glucose level was classified as normal (<109.9 mg/dL; to convert to millimoles per liter, multiply by 0.0555), impaired glucose tolerance (109.9-126.0 mg/dL), and diabetes (≥126.1 mg/dL).^34^ Contraception was defined as the use of intrauterine devices, oral contraceptives, condoms, spermicide, or other contraceptives by oneself or spouse before enrollment, regardless of duration. The number of children in the current family (including biological children, stepchildren, and adopted children) was classified as 0, 1, and 2 or more.

### Statistical Analysis

Data were analyzed between March 1, 2022, and September 30, 2022. The baseline characteristics of the study population are described by means (SD) and counts (percentages). Student *t* test and χ^2^ test were used to analyze the differences between groups. Cox proportional hazards models were used to estimate fecundability hazard ratios (HRs) and corresponding 95% CIs.^35^ HRs estimated the hazard of becoming pregnant within 1 year, and HR less than 1 indicated reduced fecundability or longer TTP.

We ran separate Cox proportional hazards models for women, men, and couples. Female model A was adjusted for the female partner’s sociodemographic characteristics, and female model B was also adjusted for the female partner’s health status, lifestyle, and menstrual and reproductive history on the basis of female model A. Similarly, male model A was adjusted for the male partner’s sociodemographic characteristics, and male model B was also adjusted for the male partner’s health status, lifestyle and number of children in the current family on the basis of male model A. The couple model was adjusted for all of the couple’s sociodemographic characteristics, health status, lifestyle, and menstrual and reproductive history. We further analyzed the interactions between each subgroup and HBV infection to explore whether the association of HBV infection with fecundability was different in subgroups. Sensitivity analysis was performed using TTP in months as outcome index to verify the robustness of the results. We also provided an assessment of missing values and participants lost to follow-up by comparing baseline characteristics. All analyses were performed with R statistical software version 1.2.5033 for Mac (R Project for Statistical Computing). Two-sided *P* < .05 was considered statistically significant.

## Results

Among these 2 419 848 couples, 126 728 women (5.24%) and 156 572 men (6.47%) were infected with HBV. The mean (SD) ages were 27.87 (5.20) for women and 29.58 (5.50) years for men. The proportions of participants with low BMI, alcohol intake, tobacco exposure, hypertension, reproductive tract infections, elevated fasting plasma glucose level, and contraception use were significantly higher in the HBV-positive group than in the HBV-negative group. The baseline characteristics of the study population are shown in Table 1.

**Table 1.** Baseline Characteristics of the Study Population

Variables	Female participants, No. (%)				Male participants, No. (%)			
	HBV negative (n = 2 293 120)	HBV positive (n = 126 728)	SMD	*P* value	HBV negative (n = 2 263 276)	HBV positive (n = 156 572)	SMD	*P* value
Sociodemographic characteristics								
Age, mean (SD), y	27.84 (5.19)	28.43 (5.38)	0.11	<.001	29.55 (5.50)	29.95 (5.52)	0.07	<.001
≤24	622 431 (27.14)	29 965 (23.65)	0.10	<.001	339 563 (15.00)	19 585 (12.51)	0.07	<.001
25-29	1 013 560 (44.20)	54 867 (43.30)			1 037 130 (45.82)	70 401 (44.96)		
30-34	390 778 (17.04)	24 114 (19.03)			497 300 (21.97)	36 983 (23.62)		
35-39	176 932 (7.72)	11 605 (9.16)			233 939 (10.34)	17 974 (11.48)		
≥40	89 419 (3.90)	6177 (4.87)			155 344 (6.86)	11 629 (7.43)		
Missing	0	0	NA	NA	0	0	NA	NA
Ethnicity								
Han	2 035 866 (89.64)	113 136 (90.58)	0.02	<.001	2 016 933 (89.99)	141 418 (91.45)	0.04	<.001
Minority ethnic groups	235 247 (10.36)	11 765 (9.42)			224 266 (10.01)	13 216 (8.55)		
Missing	22 007	1827	NA	NA	22 077	1938	NA	NA
Educational level								
Bachelor degree or above	323 393 (14.47)	21 363 (17.51)	0.07	<.001	315 164 (14.27)	26 663 (17.62)	0.08	<.001
High school or below	1 911 501 (85.53)	100 651 (82.49)			1 893 433 (85.73)	124 667 (82.38)		
Missing	58 226	4714	NA	NA	54 679	5242	NA	NA
Occupation								
Farmer	1 750 823 (78.61)	83 623 (69.12)	0.20	<.001	1 709 792 (77.77)	100 651 (67.14)	0.22	<.001
Worker	130 352 (5.85)	11 201 (9.26)			182 861 (8.32)	18 997 (12.67)		
Civil servant	130 909 (5.88)	9634 (7.96)			121 234 (5.51)	11 057 (7.38)		
Others	215 183 (9.66)	16 525 (13.66)			184 567 (8.40)	19 212 (12.82)		
Missing	65 853	5745	NA	NA	64 822	6655	NA	NA
Region								
Eastern	552 727 (24.10)	34 359 (27.11)	0.04	<.001	543 366 (24.01)	43 720 (27.92)	0.02	<.001
Central	1 162 366 (50.69)	62 893 (49.63)			1 150 401 (50.83)	74 858 (47.81)		
Western	578 027 (25.21)	29 476 (23.26)			569 509 (25.16)	37 994 (24.27)		
Missing	0	0	NA	NA	0	0	NA	NA
Health status and lifestyles								
Body mass index, mean (SD)^a^	21.81 (3.01)	21.46 (3.01)	0.12	<.001	23.34 (3.11)	23.06 (3.20)	0.09	<.001
Underweight (<18.5)	233 806 (10.21)	17 058 (13.48)	0.11	<.001	74 468 (3.29)	7210 (4.61)	0.08	<.001
Normal (18.5-23.9)	1 618 007 (70.65)	88 174 (69.69)			1 364 596 (60.38)	95 906 (61.38)		
Overweight (24.0-27.9)	347 817 (15.19)	16 903 (13.36)			648 138 (28.68)	41 796 (26.75)		
Obesity (≥28.0)	90 556 (3.95)	4388 (3.47)			172 959 (7.65)	11 328 (7.25)		
Missing	2934	205	NA	NA	3115	332	NA	NA
Alcohol intake								
Yes	36 061 (1.58)	3287 (2.60)	0.06	<.001	536 041 (22.20)	40 626 (25.99)	0.05	<.001
No	2 251 456 (98.42)	123 120 (97.40)			1 722 554 (77.80)	115 661 (74.01)		
Missing	5603	321	NA	NA	4681	285	NA	NA
Tobacco exposure								
Yes	176 361 (7.71)	11 836 (9.36)	0.04	<.001	729 270 (32.29)	57 201 (36.62)	0.09	<.001
No	2 110 596 (92.29)	114 585 (90.64)			1 528 899 (67.71)	99 017 (63.38)		
Missing	6163	307	NA	NA	5107	354	NA	NA
Hypertension								
Yes	40 537 (0.18)	2410 (0.19)	0.01	<.001	105 056 (4.66)	8071 (5.18)	0.02	<.001
No	2 243 393 (99.82)	123 760 (99.81)			2 148 882 (95.34)	147 713 (94.82)		
Missing	9190	558	NA	NA	9338	788	NA	NA
Reproductive tract infections								
Yes	41 531 (1.97)	2860 (2.55)	0.04	<.001	NA	NA	NA	NA
No	2 062 093 (98.03)	109 256 (97.45)			NA	NA	NA	NA
Missing	189 496	14 612	NA	NA	NA	NA	NA	NA
Fasting plasma glucose, mean (SD), mg/dL	88.29 (16.76)	88.65 (18.38)	0.02	<.001	NA	NA	NA	NA
<109.9	2 197 101 (96.23)	119 399 (94.69)	0.07	<.001	NA	NA	NA	NA
109.9-126.0	61 529 (2.69)	4557 (3.61)			NA	NA	NA	NA
≥126.1	24 620 (1.08)	2142 (1.70)			NA	NA	NA	NA
Missing	9870	630	NA	NA	NA	NA	NA	NA
Contraception								
Yes	747 705 (32.71)	41 465 (32.83)	<.001	.37	NA	NA	NA	NA
No	1 538 193 (67.29)	84 835 (67.17)			NA	NA	NA	NA
Missing	7222	428	NA	NA	NA	NA	NA	NA
Menstrual and reproductive history								
No. of children in current family								
0	964 488 (42.70)	54 554 (43.89)	0.02	<.001	946 285 (42.44)	72 757 (47.48)	0.10	<.001
1	1 266 131 (56.05)	68 141 (54.82)			1 255 779 (56.32)	78 493 (51.22)		
≥2	28 251 (1.25)	1594 (1.28)			27 855 (1.25)	1990 (1.30)		
Missing	34 250	2439	NA	NA	33 357	3332	NA	NA
Pregnancy history								
Yes	1 391 681 (60.72)	76 899 (60.72)	0.003	>.99	NA	NA	NA	NA
No	900 149 (39.28)	49 740 (39.28)			NA	NA	NA	NA
Missing	1290	89	NA	NA	NA	NA	NA	NA
Age at menarche, y								
<13	291 115 (12.72)	15 837 (12.53)	0.02	<.001	NA	NA	NA	NA
13-14	1 535 619 (67.08)	83 860 (66.34)			NA	NA	NA	NA
>14	462 371 (20.20)	26 719 (21.14)			NA	NA	NA	NA
Missing	4015	312	NA	NA	NA	NA	NA	NA
Menstrual period length, d								
<4.0	228 110 (9.95)	11 243 (8.88)	0.11	<.001	NA	NA	NA	NA
4.0-5.5	1 578 615 (68.89)	82 072 (64.81)			NA	NA	NA	NA
>5.5	484 925 (21.16)	33 320 (26.31)			NA	NA	NA	NA
Missing	1470	93	NA	NA	NA	NA	NA	NA
Menstrual cycle length, d								
<29	365 552 (15.94)	21 011 (16.58)	0.06	<.001	NA	NA	NA	NA
29-30	1 716 722 (74.86)	91 980 (72.58)			NA	NA	NA	NA
>30	210 846 (9.19)	13 737 (10.84)			NA	NA	NA	NA
Missing	0	0	NA	NA	NA	NA	NA	NA

Abbreviations: HBV, hepatitis B virus; NA, not applicable; SMD, standardized mean difference.

SI conversion factor: To convert glucose to millimoles per liter, multiply by 0.0555.

^a^ Body mass index is calculated as weight in kilograms divided by height in meters squared.

At the end of the 1-year follow-up period, 1650 644 couples (68.21%) were successful in getting pregnant, and the mean (SD) TTP of pregnant couples was 3.68 (3.09) months. The 1-year pregnancy rates were 65.19% among HBV-positive women (82 611 of 126 728 women), 68.38% among HBV-negative women (1 568 033 of 2 293 120 women), 66.06% among HBV-positive men (103 435 of 156 572 men), and 68.36% among HBV-negative men (1 547 209 of 2 263 276 men). This was also true when stratified by gravidity, regardless of whether women were nulligravidas (37 898 of 49 740 women [76.19%] vs 711 498 of 900 149 women [79.04%]) or multigravidas (44 649 of 76 899 women [58.06%] vs 855 554 of 1 391 681 women [61.48%]), or whether men’s female partners were nulligravidas (50 187 of 66 369 men [75.62%] vs 699 209 of 883 520 men [79.14%]) or multigravidas (53 169 of 90 089 men [59.02%] vs 847 034 of 1 378 491 men [61.45%]). Compared with the HBV-negative group, fecundability decreased by 5% (HR, 0.95; 95% CI, 0.94-0.95) in both women and men in the HBV-positive group after adjusting for potential factors. In female models, the fecundability declined by 3% (HR, 0.97; 95% CI, 0.96-0.98) among nulligravidas women and by 7% (HR, 0.93; 95% CI, 0.92-0.94) among multigravidas women. The results were similar for male models (Table 2).

**Table 2.** Association of HBV Infection With Fecundability According to the Female or Male Model^a^

HBV infection status	Female participants				Male participants			
	No.	HR (95% CI)			No.	HR (95% CI)		
		Crude	Model A	Model B		Crude	Model A	Model B
Total								
Negative	2 293 120	1 [Reference]	1 [Reference]	1 [Reference]	2 263 276	1 [Reference]	1 [Reference]	1 [Reference]
Positive	126 728	0.91 (0.91-0.92)	0.95 (0.94-0.96)	0.95 (0.94-0.95)	156 572	0.93 (0.93-0.94)	0.96 (0.96-0.97)	0.95 (0.94-0.95)
Nulligravidas women and men whose female partners were nulligravidas								
Negative	900 149	1 [Reference]	1 [Reference]	1 [Reference]	883 520	1 [Reference]	1 [Reference]	1 [Reference]
Positive	49 740	0.92 (0.91-0.93)	0.95 (0.94-0.96)	0.97 (0.96-0.98)	66 369	0.91 (0.90-0.92)	0.95 (0.95-0.96)	0.95 (0.94-0.96)
Multigravidas women and men whose female partners were multigravidas								
Negative	1 391 681	1 [Reference]	1 [Reference]	1 [Reference]	1 378 491	1 [Reference]	1 [Reference]	1 [Reference]
Positive	76 899	0.91 (0.90-0.91)	0.94 (0.93-0.95)	0.93 (0.92-0.94)	90 089	0.92 (0.91-0.93)	0.95 (0.94-0.95)	0.94 (0.93-0.95)

Abbreviations: HBV, hepatitis B virus; HR, hazard ratio.

^a^ A total of 1379 couples were not included in the analysis on the basis of gravidity owing to missing data on female partner’s pregnancy history. Female model A was adjusted for the female partner’s age (continuous), ethnicity, educational level, occupation, and region. Female model B was additionally adjusted for the female partner’s body mass index (BMI; calculated as weight in kilograms divided by height in meters squared) (continuous), alcohol intake, tobacco exposure, hypertension, reproductive tract infections, fasting plasma glucose (continuous), number of children in current family (among all women or those were multigravidas), age at menarche, menstrual period length, and menstrual cycle length based on female model A. Male model A was adjusted for the male partner’s age (continuous), ethnicity, educational level, occupation, and region. Male model B was additionally adjusted for the male partner’s BMI (continuous), alcohol intake, tobacco exposure, hypertension, and number of children in current family (among all men or those whose female partners were multigravidas) based on male model A.

In the couple models, 1-year pregnancy rates were 68.49% (1 483 430 of 2 165 932 couples) for couples in which both partners were HBV negative, 65.52% (63 779 of 97 344 couples) for couples in which the woman was HBV positive and the man was HBV negative, 66.52% (84 603 of 127 188 couples) for couples in which the woman was HBV negative and the man was HBV positive, and 64.09% (18 832 of 29 384 couples) for couples in which both partners were HBV positive. Compared with couples in which both partners were HBV negative, the fecundability of couples in which both were HBV positive declined by 6% (HR, 0.94; 95% CI, 0.93-0.96) among all couples, by 3% (HR, 0.97; 95% CI, 0.95-0.99) among nulligravidas couples, and by 7% (HR, 0.93; 95% CI, 0.91-0.95) among multigravidas couples. Therefore, the adverse association of HBV infection with fecundability was more obvious in couples with multigravidas.

**Figure 2.**
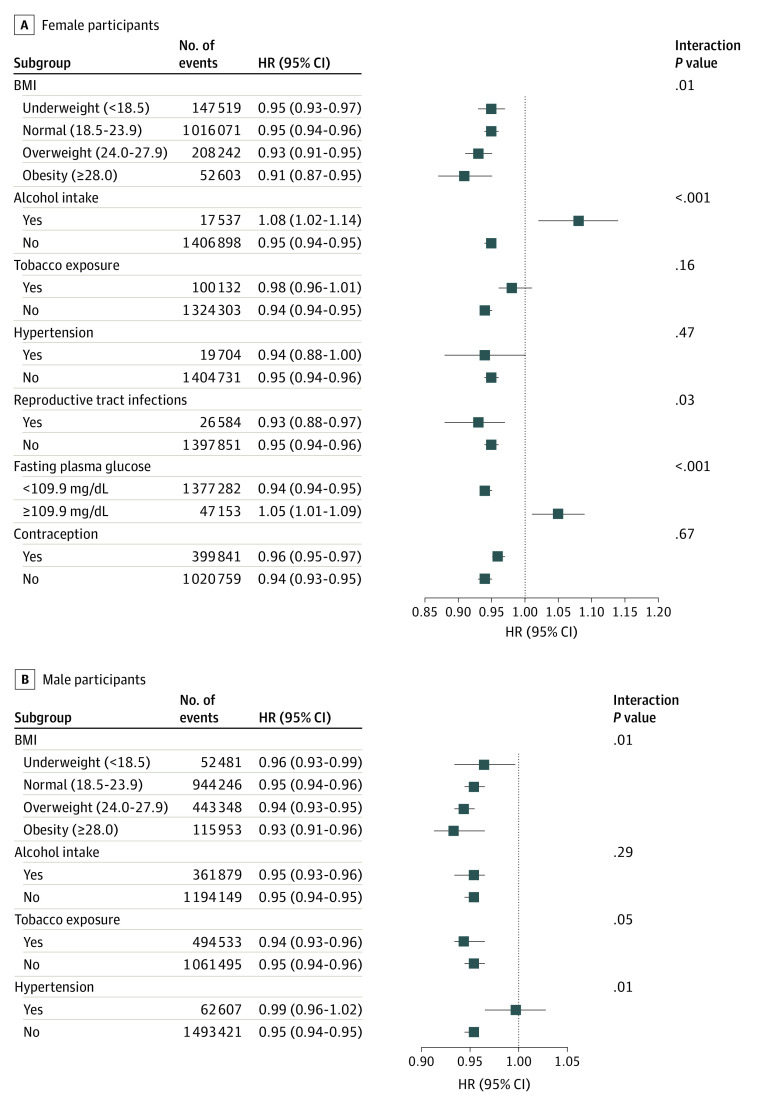
Subgroup Analysis for the Association of Hepatitis B Virus Infection With Fecundability Graphs show hazard ratios (HRs) for female partners (A) and male partners (B). Fecundability HRs for female participants were adjusted for age (continuous), ethnicity, educational level, occupation, region, body mass index (BMI; calculated as weight in kilograms divided by height in meters squared) (continuous), alcohol intake, tobacco exposure, hypertension, reproductive tract infections, fasting plasma glucose (continuous), number of children in current family, age at menarche, menstrual period length, and menstrual cycle length. HRs for male participants were adjusted for age (continuous), ethnicity, educational level, occupation, region, BMI (continuous), alcohol intake, tobacco exposure, hypertension and number of children in current family.

We further performed subgroup analyses of health status and lifestyles based on the female and male models. The results showed that the association did not appear to be modified in most subgroups. Notably, this association was more pronounced in men and women with both overweight and obesity. However, women and men had inconsistent results in certain subgroups; in particular, the negative association was more obvious in women with reproductive tract infections, normal fasting plasma glucose, and no alcohol intake, and in men with normal blood pressure (Figure 2).

Finally, the results of sensitivity analyses with TTP in months as the outcome index were consistent with the main analysis (eTable 1 in Supplement 1). Baseline characteristics of the study population vs couples excluded by missing variables or lost follow-up were detailed in eTable 2, eTable 3, and eTable 4 in Supplement 1.

## Discussion

There are more than 90 million HBV carriers in mainland China, accounting for most patients with HBV in the Asia-Pacific region,^36^ providing a large-sample population basis for this study. On the basis of this pregnancy-planning cohort, we found that HBV infection was associated with decreased fecundability, and the association was more pronounced in couples with multigravidas. Moreover, we explored whether the association varied by health statuses and lifestyles, and assessed for the first time, to our knowledge, the fecundability of reproductive-aged couples in the general population with both spouses having HBV infection, filling some shortcomings in this research field.

Previous population studies^12,13,37,38,39,40^ on the association of HBV infection with fecundability have shown inconsistent results. Studies^12,37^ of women found that HBV-infected persons undergoing assisted reproduction procedures had prolonged durations of infertility. However, a meta-analysis^38^ suggested there was no association of HBV infection with decreased fertility rate indexes, such as number of oocytes retrieved, fertilized oocytes, viable embryos, good quality follicles, pregnancy rate, or implantation rate. Most studies^39^ of men suggested that HBV infection might be related to male infertility, and HBV-infected men seeking assisted reproductive technology (ART) had poorer sperm quality, lower partner fertilization rates, and fewer embryos available for transplantation.^13,40^ It is worth mentioning that most previous relevant studies have focused on people seeking ART or those with a diagnosis of infertility, and few studies have focused on the general reproductive-aged population who are preparing for pregnancy, let alone used TTP as main indicator of fecundability.

The mechanism by which HBV infection is associated with decreased fecundability is unclear. Some studies^41,42,43^ have suggested that HBV infection is associated with immune factors that might affect female fecundability. For example, Th2 cytokines might maintain a chronic carrier state and promote fecundability, and Th1 cytokines may be associated with virus removal and inhibit fecundability, whereas a Th1/Th2 cell imbalance is thought to be a factor affecting female fecundability.^41,42,43^ Moreover, HBV infection might be closely related to Th17 cell frequency, which is associated with reduced fecundability.^44,45^ There are several hypotheses about the mechanism of HBV infection and decreased male fecundability.^46,47^ HBV might cause oxidative stress by increasing reactive oxygen species–positive cells, leading to dysfunctions including sperm membrane peroxidation and sperm motility loss. HBV infection might also cause sperm cell apoptosis and may be associated with activation of caspases 3, 8, and 9, the early indicators of apoptosis. In addition, HBV infection is closely associated with mitochondrial membrane potential loss, thus influencing the generation of reactive oxygen species, mitochondrial damage of appending sperm, energy deficiency, and cell death.

Similarly, limited previous studies^10,14,37,48^ based on a couple model only focused on the population seeking ART, and because of the low HBV infection rate in many countries, most studies defined HBV infection in couples as at least 1 partner infected, rather than both partners infected. Therefore, studies assessing fecundability in couples in which both partners have HBV infection are rare. A retrospective case-control study^37^ of 639 couples (of whom only 11 couples had dual HBV infections) found that couples in which at least 1 partner was infected with HBV had prolonged infertility, but the fecundability of couples with dual HBV infections was not studied separately. Lee et al^14^ conducted a retrospective study of 1676 couples and found no statistically significant difference in the ongoing pregnancy rate (pregnancy rate of a live fetus beyond 10 weeks’ gestation) among couples in which both partners were HBsAg positive (only 13 couples), couples with discordant HBsAg serostatus, and couples in which both partners were HBsAg negative. China has a population of 1.4 billion and a high HBV infection rate, providing a large sample population basis to assess the fecundability of reproductive-aged couples in the general population with both spouses being HBsAg positive. Because the participants were couples trying to conceive, we further conducted a stratified analysis based on gravidity, which was difficult to do in previous studies of the population seeking ART. We also unexpectedly found that the association of HBV infection with reduced fecundability was more pronounced in couples with multigravidas than in couples with nulligravidas. The associations and mechanism of HBV infection, gravidity, and fecundability remain unclear.

We also found that HBV infection was inconsistently associated with fecundability in women and men with different health statuses and lifestyles. The negative association of HBV infection with fecundability was more pronounced in women and men with overweight and obesity and in women with reproductive tract infections, suggesting that these populations should pay more attention to weight management and sexual health. Moreover, HBV infection appeared to be associated with reduced fecundability only in women with normal blood glucose and men with normal blood pressure, possibly owing to the small sample size in the hyperglycemia and hypertension subgroups; it is also possible that certain chronic conditions might modify the association. Notably, HBV infection was associated with reduced fecundability in women without tobacco exposure and women who did not use alcohol, but this negative association was found in men regardless of whether they had tobacco exposure or alcohol intake. Tobacco exposure^49^ and alcohol consumption^50^ are known to be associated with reduced fecundability in humans, but such studies have not been reported in HBV-infected people. These findings require further research to examine whether the results are reliable and explain the associations.

### Strengths and Limitations

The main advantages of our study were as follows. First, unlike previous small studies that focused on populations seeking ART, to our knowledge, this study is the first to use TTP as an indicator to investigate the association of HBV infection with fecundability in a large cohort of pregnancy-planning couples, expanding the population of such studies. Second, by use of the large sample population with HBV infection in China, to our knowledge, this study is also the first to assess the fecundability of reproductive-aged couples in the general population in which both spouses were infected with HBV; we adjusted covariates as much as possible to ensure the reliability of the results. Third, we performed stratified analyses based on gravidity, health status, and lifestyles and found that the association of HBV infection with reduced fecundability was more pronounced in couples with multigravidas, and this association varied by health status or lifestyles.

This study also had several limitations. First, the lack of time participants spent attempting pregnancy before enrollment would underestimate TTP, whereas some couples might suspend pregnancy plans during follow-up because of certain emergencies, which would overestimate TTP. Second, we lacked some data related to couples’ fecundability, such as semen quality and intercourse frequency. Third, owing to human, material, and financial constraints, we did not collect more detailed data on the HBV infection status of the participants, such as the exact time of HBV infection, HBV viral load, multiple liver function tests, HBV biopsy diagnosis, and complications or treatment information, which limited further in-depth analysis of couples with different infection status. Fourth, because the information on behavioral characteristics and menstruation in this study was self-reported, potential bias was inevitable. Fifth, there may be unmeasured or unknown potential residual confounding. Sixth, because the participants were limited to Chinese couples planning pregnancy, the results from other ethnic groups should be extrapolated with caution.

## Conclusion

On the basis of this large pregnancy-planning cohort study, we assessed for the first time the fecundability of couples in the general population with both spouses having HBV infection and found that HBV infection was associated with reduced fecundability in reproductive-aged couples. We also found that this association was more pronounced in couples with multigravidas and was inconsistent in some subgroups according to health status and lifestyle. This study supplements the research objects and results in this field and could provide references for the fertility guidance of HBV-infected couples.

## Notes

Supplement 1. **eTable 1.** Association of HBV Infection With Fecundability According to the Female or Male Model (TTP in Months)^a^ **eTable 2.** Baseline Characteristics of Couples Excluded by Irregular Menstruation or Missing Menstrual Values Versus Study Population **eTable 3.** Baseline Characteristics of Couples Excluded by Missing Testing Values of HBV Serological Indexes Versus Study Population **eTable 4.** Baseline Characteristics of Couples Who Were Recorded as Missing at the Last Follow-Up Versus Study Population

Supplement 2.
Data Sharing Statement
