# Supplementary material for: Assessment of Prior Infection With Hepatitis B Virus and Fecundability in Couples Planning Pregnancy
Source: JAMA Netw Open. 2023 Aug 31;6(8):e2330870. doi: 10.1001/jamanetworkopen.2023.30870 (PMC10472190; doi:10.1001/jamanetworkopen.2023.30870)
Supplement: Supplement 1. — eTable 1. Association of HBV Infection With Fecundability According to the Female or Male Model (TTP in Months)a eTable 2. Baseline Characteristics of Couples Excluded by Irregular Menstruation or Missing Menstrual Values Versus Study Population eTable 3. Baseline Characteristics of Couples Excluded by Missing Testing Values of HBV Serological Indexes Versus Study Population eTable 4. Baseline Characteristics of Couples Who Were Recorded as Missing at the Last Follow-Up Versus Study Population [file jamanetwopen-e2330870-s001.pdf]

Supplemental Online Content

Zhao J, Xuan Y, Zhang Y, et al. Assessment of prior infection with hepatitis B virus and fecundability in couples planning pregnancy. JAMA Netw Open. 2023;6(8):e2330870. doi:10.1001/jamanetworkopen.2023.30870

eTable 1. Association of HBV Infection With Fecundability According to the Female or Male Model (TTP in Months)[a]
eTable 2. Baseline Characteristics of Couples Excluded by Irregular Menstruation or Missing Menstrual Values Versus Study Population
eTable 3. Baseline Characteristics of Couples Excluded by Missing Testing Values of HBV Serological Indexes Versus Study Population
eTable 4. Baseline Characteristics of Couples Who Were Recorded as Missing at the Last Follow-Up Versus Study Population

This supplemental material has been provided by the authors to give readers additional information about their work.

© 2023 Zhao J et al. *JAMA Network Open.*

eTable 1 Association of HBV Infection With Fecundability According to the Female or Male Model (TTP in Months)[a]

HBV infection status	Female participants				Male participants			
	No.	Crude *HR (95% CI)*	Model A *HR (95% CI)*	Model B *HR (95% CI)*	No.	Crude *HR (95% CI)*	Model A *HR (95% CI)*	Model B *HR (95% CI)*
	**total**							
**Negative**	2,293,120	1 [Reference]	1 [Reference]	1 [Reference]	2,263,276	1 [Reference]	1 [Reference]	1 [Reference]
**Positive**	126,728	0.92 (0.91~0.92)	0.95 (0.94~0.96)	0.95 (0.94~0.96)	156,572	0.93 (0.93~0.94)	0.96 (0.96~0.97)	0.95 (0.94~0.95)
	**Nulligravidas women and men whose female partners were nulligravidas**							
**Negative**	900,149	1 [Reference]	1 [Reference]	1 [Reference]	883,520	1 [Reference]	1 [Reference]	1 [Reference]
**Positive**	49,740	0.92 (0.91~0.93)	0.95 (0.94~0.96)	0.97 (0.96~0.98)	66,369	0.91 (0.90~0.92)	0.95 (0.94~0.96)	0.95 (0.94~0.96)
	**Multigravidas women and men whose female partners were multigravidas**							
**Negative**	1,391,681	1 [Reference]	1 [Reference]	1 [Reference]	1,378,491	1 [Reference]	1 [Reference]	1 [Reference]
**Positive**	76,899	0.91 (0.90~0.92)	0.94 (0.93~0.95)	0.93 (0.93~0.94)	90,089	0.92 (0.91~0.93)	0.95 (0.94~0.95)	0.94 (0.93~0.95)

Abbreviations: HBV, hepatitis B virus; HR, hazard ratio.

[a] A total of 1379 couples were not included in the analysis on the basis of gravidity owing to missing data on female partner's pregnancy history. Female model A was adjusted for the female partner's age (continuous), ethnicity, educational level, occupation, and region. Female model B was additionally adjusted for the female partner's body mass index (BMI; calculated as weight in kilograms divided by height in meters squared) (continuous), alcohol intake, tobacco exposure, hypertension, reproductive tract infections, fasting plasma glucose (continuous), number of children in current family (among all women or those were multigravidas), age at menarche, menstrual period length, and menstrual cycle length based on female model A. Male model A was adjusted for the male partner's age (continuous), ethnicity, educational level, occupation, and region. Male model B was additionally adjusted for the male partner's BMI (continuous), alcohol intake, tobacco exposure, hypertension, and number of children in current family (among all men or those whose female partners were multigravidas) based on male model A.

© 2023 Zhao J et al. *JAMA Network Open.*

eTable 2 Baseline Characteristics of Couples Excluded by Irregular Menstruation or Missing Menstrual Values Versus Study Population

Variables	Female participants, No. (%)				Male participants, No. (%)			
	Study population (n = 2,419,848)	Couples excluded because of by irregular menstruation or missing menstrual value (n = 108,497)	SMD	*P value*	Study population (n = 2,419,848)	Couples excluded by irregular menstruation or missing menstrual value (n = 108,497)	SMD	*P value*
**Sociodemographic characteristics**								
Age, mean (SD), y	27.90 (5.20)	27.80 (5.09)	0.02	<0.001	29.60 (5.50)	29.60 (5.42)	0.002	0.40
≤24	652,396 (26.96)	29,402 (27.10)	0.03	<0.001	359,148 (14.84)	15,275 (14.08)	0.03	<0.001
25-29	1,068,427 (44.15)	48,511 (44.71)			1,107,531 (45.77)	50,522 (46.57)		
30-34	414,892 (17.15)	18,947 (17.46)			534,283 (22.08)	24,500 (22.58)		
35-39	188,537 (7.79)	7701 (7.10)			251,913 (10.41)	11,132 (10.26)		
≥40	95,596 (3.95)	3936 3.63)			166,973 (6.90)	7068 (6.51)		
Missing	0	0	NA	NA	0	0	NA	NA
Ethnicity								
Han	2,149,002 (89.69)	97,874 (91.69)	0.07	<0.001	2,158,351 (90.09)	98,467 (92.25)	0.08	<0.001
Minority ethnic groups	247,012 (10.31)	8867 (8.31)			237,482 (9.91)	8274 (7.75)		
Missing	23,834	1756	NA	NA	24,015	1756	NA	NA
Educational level								
Bachelor degree or above	344,756 (14.63)	18,946 (19.18)	0.12	<0.001	341,827 (14.48)	18,227 (18.41)	0.11	<0.001
High school or below	2,012,152 (85.37)	79,812 (80.82)			2,018,100 (85.52)	80,766 (81.59)		
Missing	62,940	9739	NA	NA	59,921	9504	NA	NA
Occupation								
Farmer	1,834,446 (78.12)	66,286 (67.53)	0.24	<0.001	1810,443 (77.09)	65,096 (66.29)	0.24	<0.001

© 2023 Zhao J et al. *JAMA Network Open.*

Worker	141,553 (6.03)	7966 (8.12)			201,858 (8.60)	12,073 (12.29)		
Civil servant	140,543 (5.99)	8900 (9.07)			132,291 (5.63)	7952 (8.10)		
Others	231,708 (9.87)	15,004 (15.29)			203,779 (8.68)	13,079 (13.32)		
Missing	71,598	10,341	NA	NA	71,477	10,297	NA	NA
Region								
Eastern	587,086 (24.26)	34,500 (31.80)	0.17	<0.001	587,086 (24.26)	34,500 (31.80)	0.17	<0.001
Central	1,225,259 (50.63)	48,495 (44.70)			1,225,259 (50.63)	48,495 (44.70)		
Western	607,503 (25.11)	25,502 (23.50)			607,503 (25.11)	25,502 (23.50)		
Missing	0	0	NA	NA	0	0	NA	NA
**Health status/lifestyles**								
Body mass index, mean (SD)[a]	21.80 (3.01)	22.1 (3.57)	0.11	<0.001	23.30 (3.11)	23.40 (3.38)	0.04	<0.001
Underweight (<18.5)	250,864 (10.38)	13,261 (12.25)	0.18	<0.001	81,678 (3.38)	4568 (4.22)	0.09	<0.001
Normal (18.5-23.9)	1,706,181 (70.60)	68,168 (62.97)			1,460,502 (60.44)	61,637 (56.96)		
Overweight (24.0-27.9)	364,720 (15.09)	19,402 (17.92)			689,934 (28.55)	31,677 (29.27)		
Obesity (≥28.0)	94,944 (3.93)	7424 (6.86)			184,287 (7.63)	10,326 (9.54)		
Missing	3139	242	NA	NA	3447	289	NA	NA
Alcohol intake								
Yes	39,348 (1.63)	3809 (3.53)	0.12	<0.001	576,667 (23.88)	35,385 (32.70)	0.20	<0.001
No	2,374,576 (98.37)	103,970 (96.47)			1,838,215 (76.12)	72,824 (67.30)		
Missing	5924	718	NA	NA	4966	288	NA	NA
Tobacco exposure								
Yes	188,188 (7.80)	15,833 (14.68)	0.22	<0.001	784,811 (32.53)	46,563 (43.06)	0.22	<0.001
No	2,225,181 (92.20)	9,2009 (85.32)			1627,916 (67.47)	61,562 (56.94)		
Missing	6479	655	NA	NA	7121	372	NA	NA
Hypertension								
Yes	42,947 (1.78)	3140 (2.91)	0.08	<0.001	113,127 (4.69)	7075 (6.56)	0.08	<0.001
No	2,367,153 (92.33)	104,687 (97.09)			2,296,595 (95.31)	100,699 (93.44)		

© 2023 Zhao J et al. *JAMA Network Open.*

Missing	9748	670	NA	NA	10,126	723	NA	NA
Reproductive tract infections								
Yes	44,391 (2.00)	2534 (2.60)	0.04	<0.001	NA	NA	NA	NA
No	2,171,349 (98.00)	95,022 (97.40)			NA	NA		
Missing	204,108	10,941	NA	NA	NA	NA	NA	NA
Fasting plasma glucose, mean (SD), mg/dL	88.29 (16.76)	89.01 (16.94)	0.04	<0.001	NA	NA	NA	NA
<109.9	2,316,500 (96.15)	103,076 (95.49)	0.03	<0.001	NA	NA	NA	NA
109.9-126.0	66,086 (2.74)	3368 (3.12)			NA	NA		
≥126.1	26,762 (1.11)	1500 (1.39)			NA	NA		
Missing	10,500	553	NA	NA	NA	NA	NA	NA
Contraception								
Yes	789,170 (32.72)	41,702 (38.80)	0.13	<0.001	NA	NA	NA	NA
No	1,623,028 (67.28)	65,769 (61.20)			NA	NA		
Missing	7650	1026	NA	NA	NA	NA	NA	NA
**Menstrual/reproduction history**								
No. of children in current family								
0	1,019,042 (42.76)	48,858 (46.62)	0.08	<0.001	1,019,042 (42.76)	48,858 (46.62)	0.08	<0.001
1	1,334,272 (55.99)	54,987 (52.67)			1,334,272 (55.99)	54,987 (52.67)		
≥2	29,845 (1.25)	954 (0.91)			29,845 (1.25)	954 (0.91)		
Missing	36,689	3698	NA	NA	36,689	3698	NA	NA
Pregnancy history								
Yes	1,468,580 (60.72)	64,548 (59.89)	0.02	<0.001	NA	NA	NA	NA
No	949,889 (39.28)	43,224 (40.11)			NA	NA		
Missing	1379	725	NA	NA	NA	NA	NA	NA
Age at menarche, y								
<13	306,952 (12.71)	13,679 (12.87)	0.12	<0.001	NA	NA	NA	NA

© 2023 Zhao J et al. *JAMA Network Open.*

13-14	1,619,479 (67.04)	65,798 (61.89)			NA	NA		
>14	489,090 (20.25)	26,845 (25.25)			NA	NA		
Missing	4327	2175	NA	NA	NA	NA	NA	NA
Menstruation Menstrual period length, d								
<4.0	239,353 (9.88)	8886 (10.93)	0.35	<0.001	NA	NA	NA	NA
4.0-5.5	1,660,687 (68.67)	43,125 (53.02)			NA	NA		
>5.5	518,245 (21.43)	29,322 (36.05)			NA	NA		
Missing	1563	27,164	NA	NA	NA	NA	NA	NA

Abbreviations: HBV, hepatitis B virus; NA, not applicable; SMD, standardized mean difference.

SI conversion factor: To convert glucose to millimoles per liter, multiply by 0.0555.

[a] Body mass index is calculated as weight in kilograms divided by height in meters squared.

© 2023 Zhao J et al. *JAMA Network Open*.

eTable 3 Baseline Characteristics of Couples Excluded by Missing Testing Values of HBV Serological Indexes Versus Study Population

Variables	Female participants, No. (%)				Male participants, No. (%)			
	Study population (n = 2,419,848)	Couples excluded by missing testing values of HBV serological indexes (n = 121,090)	SMD	*P value*	Study population (n = 2,419,848)	Couples excluded by missing testing values of HBV serological indexes (n = 121,090)	SMD	*P value*
**Sociodemographic characteristics**								
Age, mean (SD), y	27.90 (5.20)	28.90 (5.45)	0.19	<0.001	29.60 (5.50)	30.60 (5.82)	0.19	<0.001
≤24	652,396 (26.96)	25,189 (20.80)	0.17	<0.001	359,148 (14.84)	12,871 (10.63)	0.17	<0.001
25-29	1,068,427 (44.15)	51,775 (42.76)			1,107,531 (45.77)	50,665 (41.84)		
30-34	414,892 (17.15)	24,578 (20.30)			534,283 (22.08)	29,582 (24.43)		
35-39	188,537 (7.79)	13,163 (10.87)			251,913 (10.41)	16,404 (13.55)		
≥40	95,596 (3.95)	6385 (5.27)			166,973 (6.90)	11,568 (9.55)		
Missing	0	0	NA	NA	0	0	NA	NA
Ethnicity								
Han	2,149,002 (89.69)	111,762 (93.04)	0.09	<0.001	2,158,351 (90.09)	112,217 (93.32)	0.09	<0.001
Minority ethnic groups	247,012 (10.31)	8361 (6.96)			237,482 (9.91)	8035 (6.68)		
Missing	23,834	967	NA	NA	24,015	838	NA	NA
Educational level								
Bachelor degree or above	344,756 (14.63)	39,766 (33.72)	0.40	<0.001	341,827 (14.48)	38,239 (32.44)	0.38	<0.001
High school or below	2,012,152 (85.37)	78,160 (66.28)			2,018,100 (85.52)	79,645 (67.56)		
Missing	62,940	3164	NA	NA	59,921	3206	NA	NA
Occupation								
Farmer	1,834,446 (78.12)	56,823 (48.67)	0.59	<0.001	1,810,443 (77.09)	56,298 (48.21)	0.57	<0.001
Worker	141,553 (6.03)	15,199 (13.02)			201,858 (8.60)	20,139 (17.24)		

© 2023 Zhao J et al. *JAMA Network Open.*

Civil servant	140,543 (5.99)	21,061 (18.04)			132,291 (5.63)	18,257 (15.63)		
Others	231,708 (9.87)	23,667 (20.27)			203,779 (8.68)	22,091 (18.92)		
Missing	71,598	4340	NA	NA	71,477	4305	NA	NA
Region								
Eastern	587,086 (24.26)	59,900 (49.47)	0.39	<0.001	587,086 (24.26)	59,900 (49.47)	0.39	<0.001
Central	1,225,259 (50.63)	37,559 (31.02)			1,225,259 (50.63)	37,559 (31.02)		
Western	607,503 (25.11)	23,631 (19.52)			607,503 (25.11)	23,631 (19.52)		
Missing	0	0	NA	NA	0	0	NA	NA
**Health status/lifestyles**								
Body mass index, mean (SD)[a]	21.80 (3.01)	21.50 (3.06)	0.10	<0.001	23.30 (3.11)	23.50 (3.33)	0.06	<0.001
Underweight (<18.5)	250,864 (10.38)	16,767 (13.92)	0.09	<0.001	81,678 (3.38)	4913 (4.10)	0.11	<0.001
Normal (18.5-23.9)	1,706,181 (70.60)	82,593 (68.59)			1,460,502 (60.44)	66,895 (55.85)		
Overweight (24.0-27.9)	364,720 (15.09)	16,869 (14.01)			689,934 (28.55)	36,687 (30.63)		
Obesity (≥28.0)	94,944 (3.93)	4182 (3.47)			184,287 (7.63)	11,272 (9.41)		
Missing	3139	679	NA	NA	3447	1323	NA	NA
Alcohol intake								
Yes	39,348 (1.63)	6344 (5.26)	0.17	<0.001	576,667 (23.88)	40,935 (33.90)	0.21	<0.001
No	2,374,576 (98.37)	114,357 (94.74)			1,838,215 (76.12)	79,834 (66.10)		
Missing	5924	389	NA	NA	4966	321	NA	NA
Tobacco exposure								
Yes	188,188 (7.80)	19,418 (16.09)	0.21	<0.001	784,811 (32.53)	51,702 (42.82)	0.20	<0.001
No	2,225,181 (92.20)	101,274 (83.91)			1,627,916 (67.47)	69,031 (57.18)		
Missing	6479	398	NA	NA	7121	357	NA	NA
Hypertension								
Yes	42,947 (1.78)	3358 (2.80)	0.06	<0.001	113,127 (4.69)	9883 (8.33)	0.15	<0.001
No	2,367,153 (92.33)	116,450 (97.20)			2,296,595 (95.31)	108,792 (91.67)		
Missing	9748	1282	NA	NA	10,126	2415	NA	NA

© 2023 Zhao J et al. *JAMA Network Open.*

Reproductive tract infections								
Yes	44,391 (2.00)	3834 (4.25)	0.12	<0.001	NA	NA	NA	NA
No	2,171,349 (98.00)	86,361 (95.75)			NA	NA		
Missing	204,108	30,895	NA	NA	NA	NA	NA	NA
Fasting plasma glucose, mean (SD), mg/dL	88.29 (16.76)	89.55 (17.30)	0.07	<0.001	NA	NA	NA	NA
<109.9	2,316,500 (96.15)	106,942 (95.17)	0.04	<0.001	NA	NA	NA	NA
109.9-126.0	66,086 (2.74)	3856 (3.43)			NA	NA		
≥126.1	26,762 (1.11)	1567 (1.39)			NA	NA		
Missing	10,500	8725	NA	NA	NA	NA	NA	NA
Contraception								
Yes	789,170 (32.72)	58,975 (48.89)	0.32	<0.001	NA	NA	NA	NA
No	1,623,028 (67.28)	61,644 (51.11)			NA	NA		
Missing	7650	471	NA	NA	NA	NA	NA	NA
**Menstrual/reproduction history**								
No. of children in current family								
0	1,019,042 (42.76)	55,203 (46.55)	0.08	<0.001	1,019,042 (42.76)	55,203 (46.55)	0.08	<0.001
1	1,334,272 (55.99)	62,509 (52.72)			1,334,272 (55.99)	62,509 (52.72)		
≥2	29,845 (1.25)	865 (0.73)			29,845 (1.25)	865 (0.73)		
Missing	36,689	2513	NA	NA	36,689	2513	NA	NA
Pregnancy history								
Yes	1,468,580 (60.72)	72,634 (60.03)	0.001	0.69	NA	NA	NA	NA
No	949,889 (39.28)	48,360 (39.97)			NA	NA		
Missing	1379	96	NA	NA	NA	NA	NA	NA
Age at menarche, y								
<13	306,952 (12.71)	15,261 (12.64)	0.11	<0.001	NA	NA	NA	NA
13-14	1,619,479 (67.04)	76,114 (63.03)			NA	NA		

© 2023 Zhao J et al. *JAMA Network Open.*

>14	489,090 (20.25)	29,391 (24.34)			NA	NA		
Missing	4327	324	NA	NA	NA	NA	NA	NA
Menstrual period length, d								
<4.0	239,353 (9.88)	10,824 (8.94)	0.18	<0.001	NA	NA	NA	NA
4.0-5.5	1,660,687 (68.67)	70,875 (58.57)			NA	NA		
>5.5	518,245 (21.43)	39,317 (32.49)			NA	NA		
Missing	1563	74	NA	NA	NA	NA	NA	NA
Menstrual cycle length, d								
<29	386,563 (15.99)	22,850 (18.87)	0.12	<0.001	NA	NA	NA	NA
29-30	1,808,702 (74.79)	83,713 (69.13)			NA	NA		
>30	224,583 (9.29)	14,527 (12.00)			NA	NA		
Missing	0	0	NA	NA	NA	NA	NA	NA

Abbreviations: HBV, hepatitis B virus; NA, not applicable; SMD, standardized mean difference.

SI conversion factor: To convert glucose to millimoles per liter, multiply by 0.0555.

[a] Body mass index is calculated as weight in kilograms divided by height in meters squared.

© 2023 Zhao J et al. *JAMA Network Open*.

eTable 4 Baseline Characteristics of Couples Who Were Recorded as Missing at the Last Follow-Up Versus Study Population

Variables	Female participants, No. (%)				Male participants, No. (%)			
	Study population (n = 2,419,848)	Couples who were recorded as missing at the last follow-up (n = 6202)	SMD	*P value*	Study population (n = 2,419,848)	Couples who were recorded as missing at the last follow-up (n = 6202)	SMD	*P value*
**Sociodemographic characteristics**								
Age, mean (SD), y	27.90 (5.20)	27.70 (5.56)	0.03	<0.001	29.60 (5.50)	30.10 (5.81)	0.09	<0.001
≤24	652,396 (26.96)	1943 (31.33)	0.12	<0.001	359,148 (14.84)	897 (14.46)	0.10	<0.001
25-29	1,068,427 (44.15)	2433 (39.23)			1,107,531 (45.77)	2615 (42.16)		
30-34	414,892 (17.15)	1002 (16.16)			534,283 (22.08)	1451 (23.40)		
35-39	188,537 (7.79)	534 (8.61)			251,913 (10.41)	714 (11.51)		
≥40	95,596 (3.95)	290 (4.68)			166,973 (6.90)	525 (8.47)		
Missing	0	0	NA	NA	0	0	NA	NA
Ethnicity								
Han	2,149,002 (89.69)	4462 (72.78)	0.47	<0.001	2,158,351 (90.09)	4456 (72.67)	0.49	<0.001
Minority ethnic groups	247,012 (10.31)	1669 (27.22)			237,482 (9.91)	1676 (27.33)		
Missing	23,834	71	NA	NA	24,015	70	NA	NA
Educational level								
Bachelor degree or above	344,756 (14.63)	933 (15.63)	<0.001	>.99	341,827 (14.48)	913 (15.29)	0.01	0.65
High school or below	2,012,152 (85.37)	5036 (84.37)			2,018,100 (85.52)	5059 (84.71)		
Missing	62,940	233	NA	NA	59,921	230	NA	NA
Occupation								
Farmer	1,834,446 (78.12)	4224 (71.46)	0.15	<0.001	1,810,443 (77.09)	4168 (70.60)	0.14	<0.001
Worker	141,553 (6.03)	555 (9.39)			201,858 (8.60)	716 (12.13)		
Civil servant	140,543 (5.99)	467 (7.90)			132,291 (5.63)	422 (7.15)		

© 2023 Zhao J et al. *JAMA Network Open.*

Others	231,708 (9.87)	665 (11.25)			203,779 (8.68)	598 (10.13)		
Missing	71,598	291	NA	NA	71,477	298	NA	NA
Region								
Eastern	587,086 (24.26)	1235 (19.91)	0.99	<0.001	587,086 (24.26)	1235 (19.91)	0.99	<0.001
Central	1,225,259 (50.63)	925 (14.91)			1,225,259 (50.63)	925 (14.91)		
Western	607,503 (25.11)	4042 (65.17)			607,503 (25.11)	4042 (65.17)		
Missing	0	0	NA	NA	0	0	NA	NA
**Health status/lifestyles**								
Body mass index, mean (SD)[a]	21.80 (3.01)	21.60 (3.02)	0.06	<0.001	23.30 (3.11)	23.00 (3.07)	0.11	<0.001
Underweight (<18.5)	250,864 (10.38)	774 (12.51)	0.06	<0.001	81,678 (3.38)	270 (4.36)	0.10	<0.001
Normal (18.5-23.9)	1,706,181 (70.60)	4257 (68.83)			1,460,502 (60.44)	3947 (63.74)		
Overweight (24.0-27.9)	364,720 (15.09)	942 (15.23)			689,934 (28.55)	1575 (25.44)		
Obesity (≥28.0)	94,944 (3.93)	212 (3.43)			184,287 (7.63)	400 (6.46)		
Missing	3139	17	NA	NA	3447	10	NA	NA
Alcohol intake								
Yes	39,348 (1.63)	157 (2.54)	0.05	<0.001	576,667 (23.88)	1921 (31.04)	0.16	<0.001
No	2,374,576 (98.37)	6030 (97.46)			1,838,215 (76.12)	4267 (68.96)		
Missing	5924	15	NA	NA	4966	14	NA	NA
Tobacco exposure								
Yes	188,188 (7.80)	534 (8.64)	0.01	0.49	784,811 (32.53)	2506 (40.45)	0.15	<0.001
No	2,225,181 (92.20)	5643 (91.36)			1,627,916 (67.47)	3689 (59.55)		
Missing	6479	25	NA	NA	7121	7	NA	NA
Hypertension								
Yes	42,947 (1.78)	96 (1.56)	0.02	0.18	113,127 (4.69)	279 (4.54)	0.01	0.42
No	2,367,153 (92.33)	6053 (98.44)			2,296,595 (95.31)	5873 (95.46)		
Missing	9748	53	NA	NA	10,126	50	NA	NA
Reproductive tract infections								

© 2023 Zhao J et al. *JAMA Network Open.*

Yes	44,391 (2.00)	126 (2.35)	0.02	0.18	NA	NA	NA	NA
No	2,171,349 (98.00)	5242 (97.65)			NA	NA		
Missing	204,108	834	NA	NA	NA	NA	NA	NA
Fasting plasma glucose, mean (SD), mg/dL	88.29 (16.76)	88.83 (19.64)	0.03	0.07	NA	NA	NA	NA
<109.9	2,316,500 (96.15)	5807 (94.35)	0.09	<0.001	NA	NA	NA	NA
109.9-126.0	66,086 (2.74)	225 (3.66)			NA	NA		
≥126.1	26,762 (1.11)	123 (2.00)			NA	NA		
Missing	10,500	47	NA	NA	NA	NA	NA	NA
Contraception								
Yes	789,170 (32.72)	2156 (34.95)	0.06	<0.001	NA	NA	NA	NA
No	1,623,028 (67.28)	4012 (65.05)			NA	NA		
Missing	7650	34	NA	NA	NA	NA	NA	NA
**Menstrual/reproduction history**								
No. of children in current family								
0	1,019,042 (42.76)	2751 (45.23)	0.20	<0.001	1,019,042 (42.76)	2751 (45.23)	0.20	<0.001
1	1,334,272 (55.99)	3085 (50.72)			1,334,272 (55.99)	3085 (50.72)		
≥2	29,845 (1.25)	246 (4.04)			29,845 (1.25)	246 (4.04)		
Missing	36,689	120	NA	NA	36,689	120	NA	NA
Pregnancy history								
Yes	1,468,580 (60.72)	3758 (60.61)	0.05	<0.001	NA	NA	NA	NA
No	949,889 (39.28)	2442 (39.39)			NA	NA		
Missing	1379	2	NA	NA	NA	NA	NA	NA
Age at menarche, y								
<13	306,952 (12.71)	741 (11.98)	0.07	<0.001	NA	NA	NA	NA
13-14	1,619,479 (67.04)	4036 (65.24)			NA	NA		
>14	489,090 (20.25)	1409 (22.78)			NA	NA		

© 2023 Zhao J et al. *JAMA Network Open.*

Missing	4327	16	NA	NA	NA	NA	NA	NA
Menstruation Menstrual period length, d								
<4.0	239,353 (9.88)	906 (14.61)	0.15	<0.001	NA	NA	NA	NA
4.0-5.5	1,660,687 (68.67)	4041 (65.19)			NA	NA		
>5.5	518,245 (21.43)	1252 (20.20)			NA	NA		
Missing	1563	3	NA	NA	NA	NA	NA	NA
Period Menstrual cycle length, d								
<29	386,563 (15.99)	1035 (16.69)	0.03	0.09	NA	NA	NA	NA
29-30	1,808,702 (74.79)	4505 (72.64)			NA	NA		
>30	224,583 (9.29)	662 (10.67)			NA	NA		
Missing	0	0	NA	NA	NA	NA	NA	NA

Abbreviations: HBV, hepatitis B virus; NA, not applicable; SMD, standardized mean difference.

SI conversion factor: To convert glucose to millimoles per liter, multiply by 0.0555.

[a] Body mass index is calculated as weight in kilograms divided by height in meters squared.

© 2023 Zhao J et al. *JAMA Network Open.*
