# Supplementary material for: Assessment of Prior Infection With Hepatitis B Virus and Fecundability in Couples Planning Pregnancy
Source: JAMA Netw Open. 2023 Aug 31;6(8):e2330870. doi: 10.1001/jamanetworkopen.2023.30870 (PMC10472190; doi:10.1001/jamanetworkopen.2023.30870)
Supplement: Supplement 2. — Data Sharing Statement [file jamanetwopen-e2330870-s002.pdf]

Data Sharing Statement

Zhao. Assessment of Prior Infection With Hepatitis B Virus and Fecundability in Couples Planning Pregnancy. *JAMA Netw Open*. Published August 31, 2023. doi:10.1001/jamanetworkopen.2023.30870

Data
Data available: No

Additional Information
Explanation for why data not available: The National Free Pre-conception Check-up Project data contains sensitive data and cannot be shared via public deposition because of information governance restrictions in place to protect individuals' confidentiality. Access to data for external researchers (not affiliated with the National Research Institute for Family Planning) requires researchers to be physically based in the institute. Access to data is available only once approval has been obtained through the individual constituent entities controlling access to the data.
